# A human stool-derived *Bilophila wadsworthia* strain caused systemic inflammation in specific-pathogen-free mice

**DOI:** 10.1186/s13099-017-0208-7

**Published:** 2017-10-26

**Authors:** Zhou Feng, Wenmin Long, Binhan Hao, Ding Ding, Xiaoqing Ma, Liping Zhao, Xiaoyan Pang

**Affiliations:** State Key Laboratory of Microbial Metabolism, School of Life Sciences and Biotechnology, Shanghai Jiao Tong University, 800 Dongchuan Road, Minhang District, Shanghai, 200240 China

**Keywords:** *Bilophila wadsworthia*, Inflammation, Hepatosplenomegaly, Gut microbiota, Mice

## Abstract

**Background:**

*Bilophila wadsworthia* is a major member of sulfidogenic bacteria in human gut, it was originally recovered from different clinical specimens of intra-abdominal infections and recently was reported potentially linked to different chronic metabolic disorders. However, there is still insufficient understanding on its detailed function and mechanism to date.

**Methods:**

A *B. wadsworthia* strain was isolated from fresh feces of a latent autoimmune diabetes in adults patient and we investigated its pathogenicity by oral administration to specific-pathogen-free mice. Tissue samples and serum were collected after sacrifice. Stool samples were collected at different time points to profile the gut microbiota.

**Results:**

*Bilophila wadsworthia* infection resulted in the reduction of body weight and fat mass, apparent hepatosplenomegaly and elevated serum inflammatory factors, including serum amyloid A and interleukin-6, while without significant change of the overall gut microbiota structure.

**Conclusions:**

These results demonstrated that higher amount of *B. wadsworthia* caused systemic inflammatory response in SPF mice, which adds new evidence to the pathogenicity of this bacterium and implied its potential role to the chronic inflammation related metabolic diseases like diabetes.

## Background

Gut microbiota has been closely linked with many chronic and refractory diseases such as inflammatory bowel disease, colorectal cancer, obesity, diabetes, and even mental diseases like autism. The key roles of an increasing number of gut bacteria have been revealed, which herald a great progress in our knowledge of the etiology of those diseases and offer great promise for optimizing health and treating diseases in novel ways. For instance, an *Enterobacter cloacae* strain has been identified as an obesity-inducing opportunistic pathogen, whose mono-association in germfree mice can recapitulate the obese phenotype, including low-grade inflammation, adiposity and insulin resistance [1]. Another bacterium, *Akkermansia muciniphila* has been demonstrated to negatively correlated with symptoms of obesity and type 2 diabetes, and has the potential as a probiotic to reverse high-fat diet-induced metabolic disorders in mice [2]. Recent advances in microbial cultivation and model animal are greatly facilitating our understanding of the fundamental and ultimate question—”who” does “what” and “how” referring to the active roles and mechanisms of human gut microbiota [3].

*Bilophila wadsworthia* is a sulfite-reducing and hydrogen sulfide-producing microbe, which was originally isolated from specimens of peritoneal fluid and tissue of patients with appendicitis [4], while usually difficult to detect in healthy individuals. *B. wadsworthia* is an obligately anaerobic Gram-negative bacillus and can be stimulated by bile. Since it was named in 1989, there has been limited progress on this bacterium. Till in 2012, researchers began to realize the function of this commensal gut bacterium. Devkota reported that a milk-derived saturated fat diet induced a bloom of *B. wadsworthia* in gut of SPF mice. And mono-inoculation of this pathobiont in germfree IL10^−/−^ mice fed with milk fat diet can even induce T_H_1 immune response and colitis development [5]. This bacterium was also detected over-represented in colonic microbiota of colorectal cancer patients, which implied its possible role in colorectal carcinogenesis [6]. Though mounting studies have highlighted the correlations of *B. wadsworthia* in different human diseases [7–9], especially chronic metabolic diseases, the mechanisms of its pathogenicity are not yet well characterized.

In the study described here, we obtained an isolate of *B. wadsworthia* from a new-onset LADA (latent autoimmune diabetes in adults) patient. The objective of this study was to examine the outcome of the infection of this strain in normal SPF mice, and try to elicit its possible pathogenicity.

## Methods

### Isolation and identification of *B. wadsworthia*

The *B. wadsworthia* strain used in this study was isolated from fresh fecal samples of a newly diagnosed LADA 30-year-old female patient. The study was approved by Ethics Committee at Shanghai General Hospital, School of Medicine, Shanghai Jiao Tong University. Informed consent was signed by the patient. By coincidence, we had already collected and stored two fecal samples of this individual 4 years ago when there wasn’t any LADA related symptom developed in this girl. The DNA abundance of *B. wadsworthia* before and after LADA onset was detected by Real-time PCR using SYBR Green Supermix (Bio-Rad, 170-8882AP). DNA extraction from fecal samples was conducted as previously described [10]. A *B. wadsworthia*-specific PCR primer set targeted taurine:pyruvate aminotransferase (*Tpa*) gene was used to quantify *B. wadsworthia* in stool [11], and primer set Uni331-F/Uni797-R targeted universal bacterial 16S rRNA gene was used to quantify total bacteria [12].

For *B. wadsworthia* isolation, a fresh fecal sample was collected and transported into the anaerobic workstation (DG500, DWS, UK) within 5 min after defecation. Bacteria were isolated using Postgate E medium [13] supplemented with 0.1% (w/v) bile salt, and then transferred to ABB medium (Anaerobe basal broth) [14] supplemented with 10 mM taurine for rapid growth. Identification of *B. wadsworthia* was confirmed by 16S rRNA gene sequencing.

To easily detect this *B. wadsworthia* strain in fecal samples of subsequent mice experiment, a spontaneous mutant strain for rifampicin resistance was selected on rifampicin-containing media [15] and was used as the inoculum to mice.

### Animal trial

Twenty 6-week-old male SPF C57BL/6 mice were purchased from SLAC Inc. (Shanghai, China). Mice were maintained under a regular 12-h light cycle and fed with normal chow diet ad libitum. After 2 weeks acclimation, animals were randomly assigned to either BW group (daily gavaged with 1 × 10^8^ CFU *B. wadsworthia* suspended in 100 μL anaerobic PBS solution for 1 week) or NC group (daily gavaged with an equivalent volume of anaerobic PBS solution for 1 week). Body weight and food intake were recorded every day. Fresh fecal samples were collected on Day 0, 1, 5 and 7. Living cells of *B. wadsworthia* were detected by plate counting on ABB agar supplemented with 10 mM taurine and 100 μg/mL rifampicin. After sacrifice on Day 7, blood, liver, spleen, fat and colon were collected. Distal colons were fixed in 4% paraformaldehyde, embedded in paraffin, and stained with hematoxylin/eosin. The ethical treatment of animals was assured by the Animal Care and Use Committee of the School of Life Sciences and Biotechnology, Shanghai Jiao Tong University.

### Enzyme-linked immunosorbent assay (ELISA)

Serum concentrations for the following inflammation markers were determined using ELISA following the manufacturer’s instructions: Serum Amyloid A (SAA, Tridelta, TP802-M), Lipopolysaccharide Binding Protein (LBP, Cell Sciences, CKM043), Interleukin-6 (IL-6, eBioscience, BMS603HS) and tumor necrosis factor-alpha (TNF-α, eBioscience, BMS607HS).

### Detection of gene expression in the colon

Total RNA from colon was isolated using RNeasy Mini Kit (QIAGEN, 74106). 1 μg of RNA was treated with RNase-free DNase I (Invitrogen, 18068-015). cDNA was synthesized using SuperScript III First-Strand Synthesis System for RT-PCR kit (Invitrogen, 18080-051). qPCR was performed using SYBR Green Supermix (Bio-Rad, 170-8882AP). Glyceraldehyde-3-phosphate dehydrogenase (*Gapdh*) gene was used as an endogenous housekeeping gene control. Relative gene expression was determined using the 2^−ΔΔCt^ method [16]. Gene expression levels were normalized to that of NC group. Primer sequences for the targeted mouse genes were presented in Table 1 [17]:

**Table 1:** Primers used in quantitative PCR assays

Primers	Sequences
Gapdh-F	5′-GTGTTCCTACCCCCAATGTGT-3′
Gapdh-R	5′-ATTGTCATACCAGGAAATGAGCTT-3′
Il6-F	5′-GTTCTCTGGGAAATCGTGGA-3′
Il6-R	5′-TGTACTCCAGGTAGCTA-3′
Tnfα-F	5′-ACGGCATGGATCTCAAAGAC-3′
Tnfα-R	5′-AGATAGCAAATCGGCTGACG-3′
Tlr4-F	5′-ATGGCATGGCTTACACCACC-3′
Tlr4-R	5′-GAGGCCAATTTTGTCTCCACA-3′

### Gut microbiota profiling

DNA extraction from fecal samples was conducted as previously described [18]. A sequencing library of V3–V4 region of the bacterial 16S rRNA gene was prepared following the manufacturer’s instruction with some modifications [19]. The locus-specific primers for V3–V4 region of the bacterial 16S rRNA gene are as follows: Forward Primer: 5′-CCTACGGGNGGCWGCAG-3′; Reverse Primer: 5′-GACTACHVGGGTATCTAATCC-3′. For the Amplicon PCR step (amplification of 16S rRNA gene V3–V4 region), the 25 μL reaction mix consisted of 1 × Phanta Max Buffer, 0.2 mM dNTP, 0.25 μM Forward Primer, 0.25 μM Reverse Primer, 0.5 U of Phanta Max Super-Fidelity DNA Polymerase and 10 ng template DNA. The program consisted of 1 cycle of 95 °C for 3 min, followed by 21 cycles of 95 °C for 30 s, 55 °C for 30 s and 72 °C for 30 s, and finally 1 cycle of 72 °C for 5 min. For the Index PCR step (attachment of Illumina sequencing adapters and dual-index barcodes), the 25 μL reaction mix consisted of 1 × Phanta Max Buffer, 0.2 mM dNTP, 2.5 μL Nextera XT Index Primer 1 (N7XX), 2.5 μL Nextera XT Index Primer 2 (S5XX), 0.5 U of Phanta Max Super-Fidelity DNA Polymerase and 2.5 μL purified product of the Amplicon PCR step as template DNA. The program consisted of 1 cycle of 95 °C for 3 min, followed by 8 cycles of 95 °C for 30 s, 55 °C for 30 s and 72 °C for 30 s, and finally 1 cycle of 72 °C for 5 min. The purified products were mixed at equal ratio for sequencing on Illumina Miseq platform (Illumina Inc., USA).

After sequencing, high quality sequences were selected for further analysis. Operational Taxonomic Units (OTUs) were picked by Usearch (v8.0.1623_i86linux32) at 97% cutoff [20]. Alpha-diversity (including observed OTUs and Shannon index) and beta-diversity (based on bray–curtis distance, unweighted-unifrac distance and weighted-unifrac distance) were performed on QIIME platform [21].

### Statistical analysis

Statistical analysis was performed with Statistical Package for Social Sciences software version 23.0 (SPSS Inc., USA). To detect the variation between NC group and BW group, independent samples *t* test or Mann–Whitney U test was used, depending on whether the data were normally distributed or not.

## Results

### Isolation and identification of *B. wadsworthia*

The *B. wadsworthia* strain was isolated from fresh feces of a female volunteer who was newly diagnosed with LADA. We compared the abundance of *B. wadsworthia* in fecal samples of this individual before and after the onset of LADA. In the acute disease stage, the abundance of *B. wadsworthia* was considerably higher than the sample collected 4 years before the diagnosis (1.11 × 10^3^ vs. 1.35 × 10^2^ copies/ng fecal DNA). After modification of *tpa* gene copy number with 16S rRNA gene copy number, the relative abundance of *B. wadsworthia* changed from 0.003 to 0.017% with the onset of LADA.

**Fig. 1:**
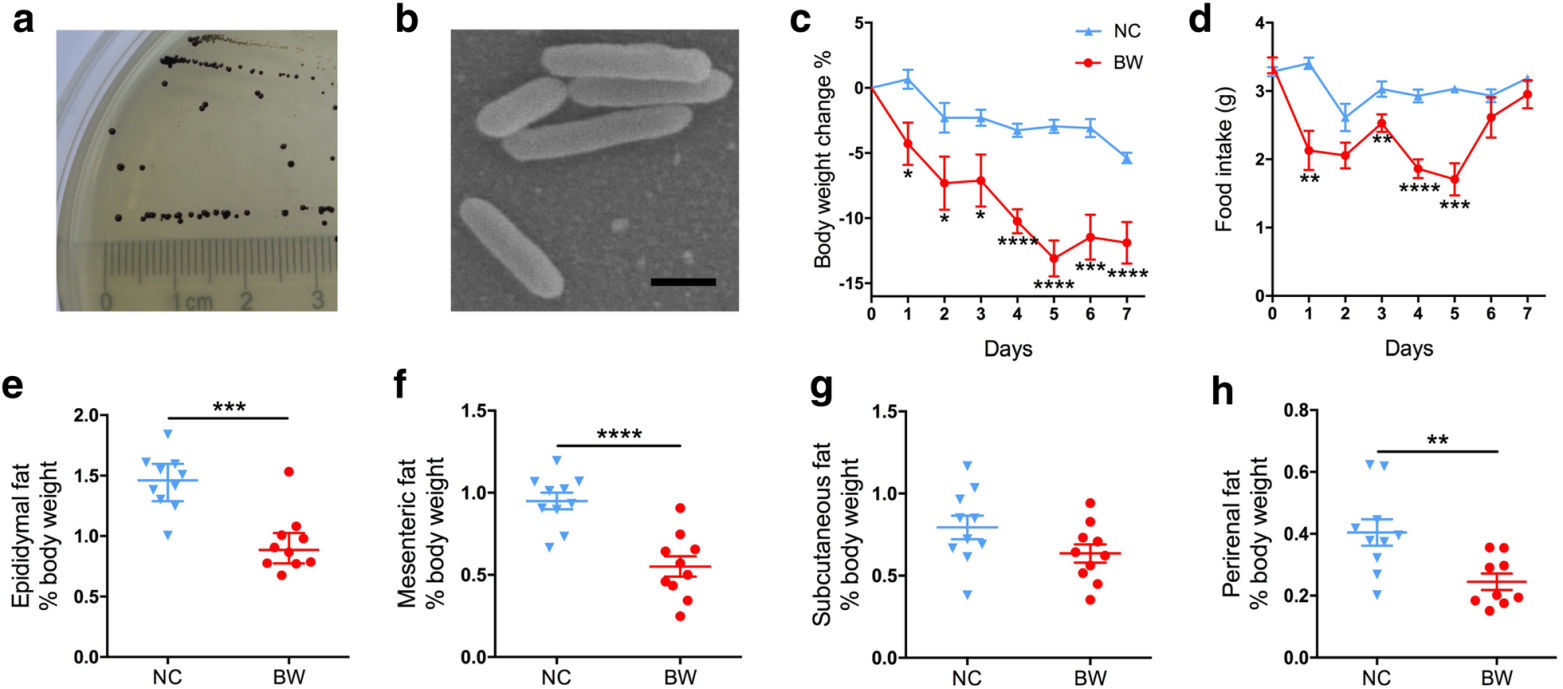
Morphology of *B. wadsworthia* and its effects in reducing body weight and fat mass of mice. **a** Colonies of *B. wadsworthia* on ABB agar after 2 days at 37 °C. **b** Electron micrograph of *B. wadsworthia*. Scale bar, 1 μm. Changes of body weight (**c**), food intake (**d**), mass of epididymal fat (**e**), mesenteric fat (**f**), subcutaneous fat (**g**) and perirenal fat (**h**) of NC and BW group. For **c**–**g**, data are shown as mean ± S.E.M. and tested by independent samples *t* test. For **e**, data are shown as median with interquartile range and tested by Mann–Whitney U test. *P < 0.05, **P < 0.01, ***P < 0.001, ****P < 0.0001. For **c**–**g**, both groups n = 10; for **h**, NC group n = 10, BW group n = 9

The strain was first isolated using Postgate E medium supplemented with bile salts, and then transferred to ABB supplemented with taurine. On ABB agar, colonies of *B. wadsworthia* appeared circular, black or translucent with dark black centers, low convex, with a diameter of about 0.5–1 mm after 2 days at 37 °C in anaerobic workstation (Fig. 1a). The cells were rods and no flagella or other appendages were observed under electron microscopy (Fig. 1b). Its 16S rRNA gene sequence showed 99% identity with *B. wadsworthia* Marseille-AA00033.

### *B. wadsworthia* infection reduced the body weight and fat mass

To characterize the effect of *B. wadsworthia* infection, we orally inoculated SPF mice for 7 days continuously, with 1 × 10^8^ CFU per day. The target strain was detected in stool samples of each mouse at the end of the trial with the abundance ranging from 2.36 × 10^3^ to 2.45 × 10^6^ CFU/g of feces.

Following oral inoculation with *B. wadsworthia*, the body weight of mice in BW group decreased compared with NC group. On Day 5, the body weight of BW mice reduced to the lowest level with 13.1% loss on average, and at the last day of the experiment (Day 7) with 11.9% loss on average (Fig. 1c). Monitoring of food intake showed that BW mice also decreased their food intake markedly before Day 5, while in the last 2 days, food intake of this group recovered to the similar level with that of NC group (Fig. 1d).

We also measured the major fat mass of both groups. In BW mice, a significant reduction of the mass of epididymal, mesenteric and perirenal fat was observed, with 35.1, 42.0 and 39.4% loss on average respectively, compared with NC group. Subcutaneous fat mass displayed a decreased tendency but not statistically significant (Fig. 1e–h).

### *B. wadsworthia* infection induced hepatosplenomegaly

**Fig. 2:**
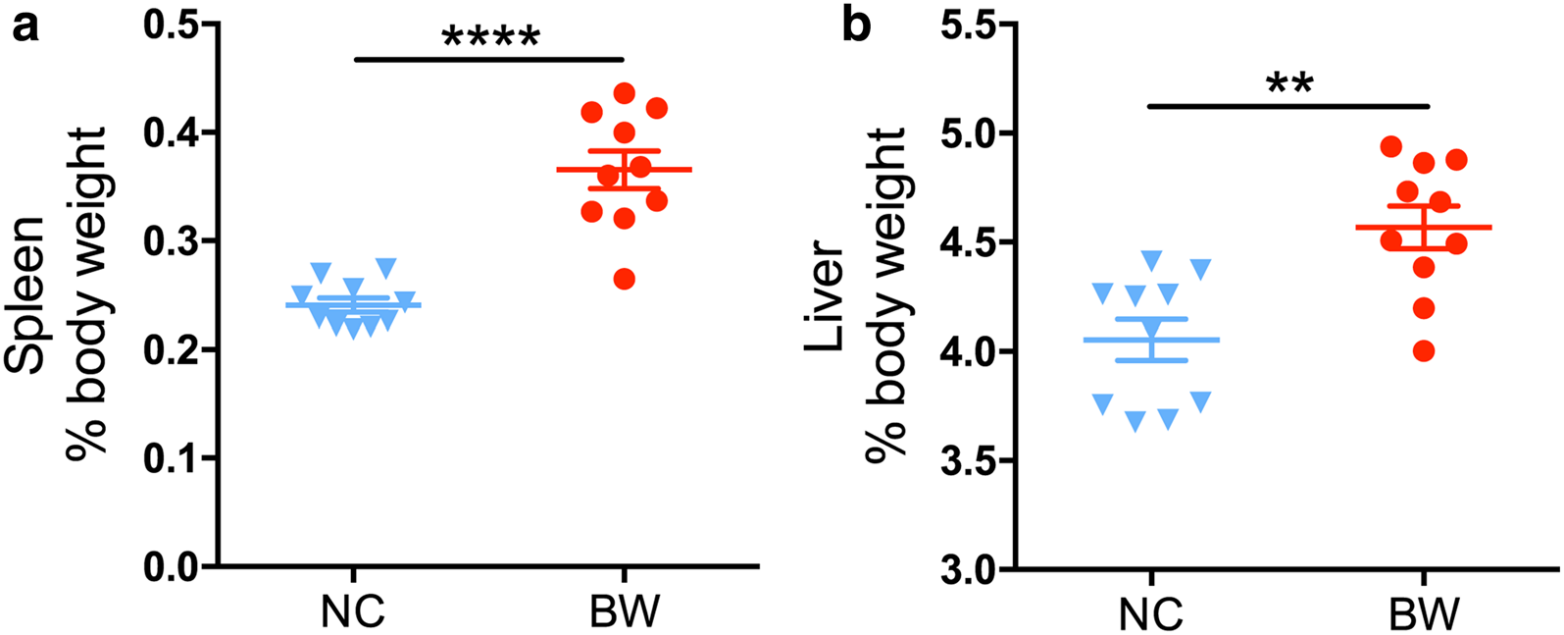
Weight of spleen (**a**) and liver (**b**) for each group (n = 10). Data are shown as mean ± S.E.M. and test by independent samples *t* test. **P < 0.01, ****P < 0.0001

Gross examination of *B. wadsworthia* infected mice at the time of necropsy revealed nearly no noticeable lesions in organs. While when the whole spleen and liver were weighed, it was observed that inoculation of *B. wadsworthia* resulted in marked splenomegaly and enlarged liver on Day 7. The spleen: body weight ratio of BW group mice significantly higher than that of NC group (*P* < 0.0001), and the same with the liver: body weight ratio comparison (*P* < 0.01) (Fig. 2).

### *B. wadsworthia* infection didn’t induce aberrant changes of colon

**Fig. 3:**
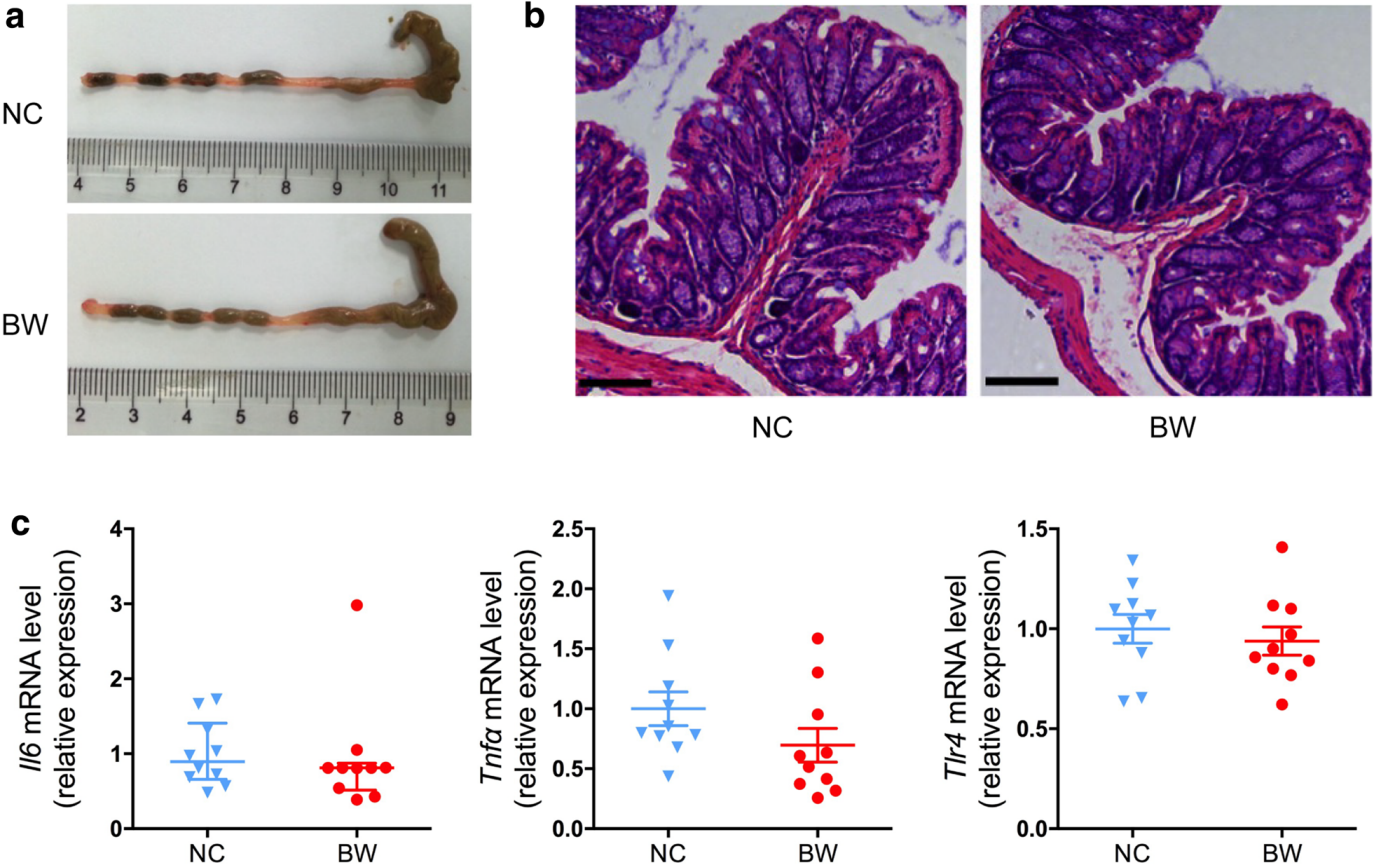
Comparison of colon in mice of two groups. **a** Representative colon lengths in mice. **b** Haematoxylin–eosin staining of distal colon. Scale bars, 100 μm. **c** RT-qPCR analysis of expression of *Il6*, *Tnfα* and *Tlr4* in the colon (n = 10). For *Il6*, data are shown as median with interquartile range and tested by Mann–Whitney U test. For *Tnfα* and *Tlr4*, data are shown as mean ± S.E.M. and tested by independent samples *t* test

Compared with NC group, the length and morphology of colon in mice treated with *B. wadsworthia* didn’t exhibit significant changes (Fig. 3a, b). And the relative gene expression of pro-inflammatory cytokines IL-6, TNF-α and Toll-like Receptor-4 (TLR-4) in colon in the two groups didn’t show statistical difference, which implied that *B. wadsworthia* didn’t induce local inflammation in colon (Fig. 3c).

### *B. wadsworthia* induced overrepresented systemic inflammation

**Fig. 4:**
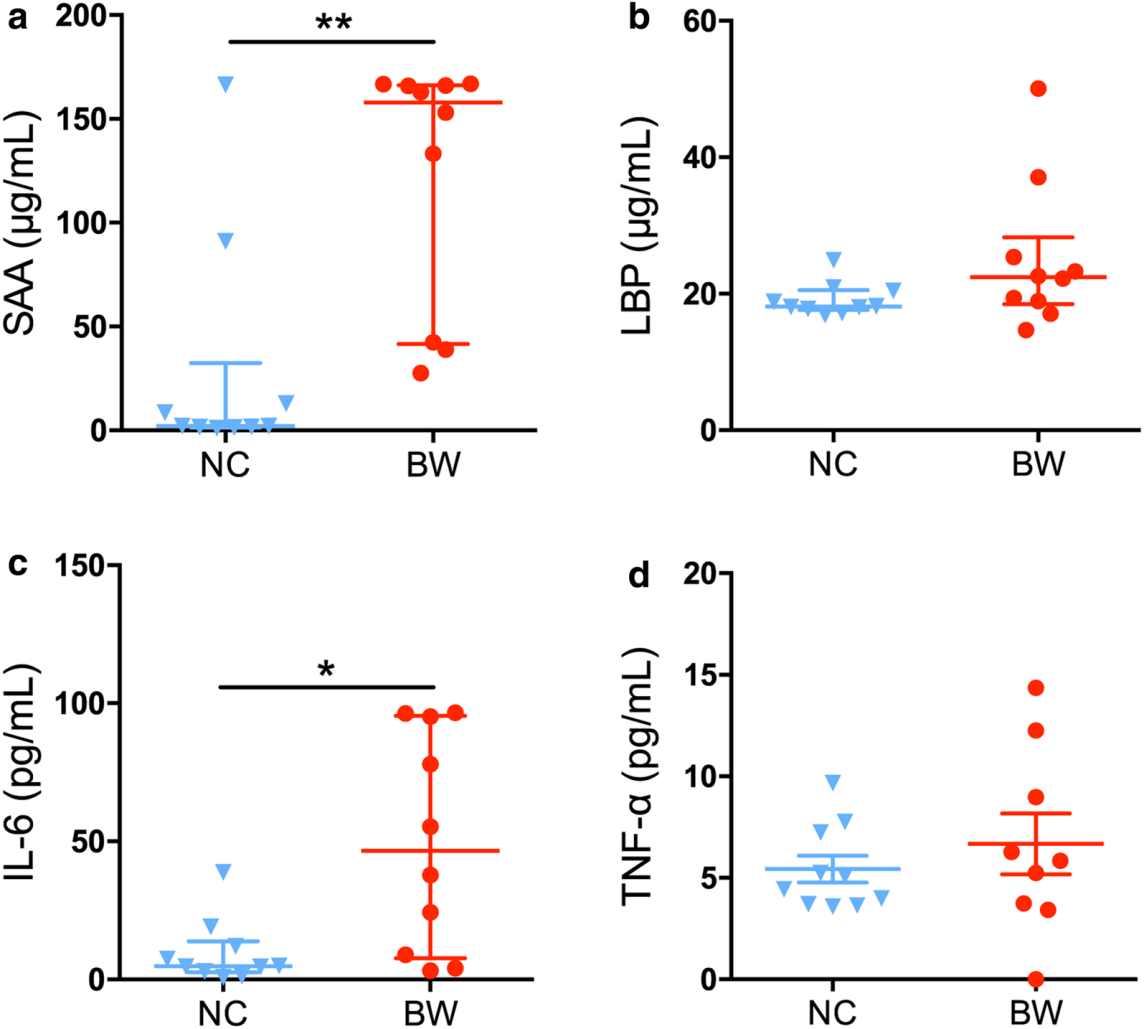
ELISA analysis of Serum Amyloid A (**a**), LBP (**b**), IL-6 (**c**) and TNF-α (**d**). For **a**–**c**, data are shown as median with interquartile range and tested by Mann–Whitney U test, both groups n = 10. For **d**, data are shown as mean ± S.E.M. and tested by independent samples *t* test, NC group n = 10, BW group n = 9. *P < 0.05, **P < 0.01

Enzyme-linked immunosorbent assay analysis of serum inflammation indexes showed that *B. wadsworthia* infection significantly increased the SAA level (*P* < 0.01) of mice. Serum LBP of this group tended to be higher, but the difference was not statistically significant. In BW mice, the pro-inflammatory cytokine IL-6 levels were also higher than those in the NC group (*P* < 0.05), while TNF-α level was not significantly upregulated than NC group (*P* > 0.05) (Fig. 4).

### *B. wadsworthia* inoculation did not significantly change the gut microbiota of mice

To profile the overall structure of gut microbiota after *B. wadsworthia* gavage, the V3–V4 region of the bacterial 16S rRNA gene was sequenced for fecal samples of mice in both groups on Day 0, 1, 5 and 7, using Illumina Miseq platform. A total of 613,626 quality-filtered reads were obtained. The average reads number per sample was 15,341 ± 1628 (mean ± S.D.), with the highest reads number 18,813 and lowest 10,822, which implied a uniform sequence distribution. Reads were classified into 539 OTUs at 97% similarity level with Usearch.

**Fig. 5:**
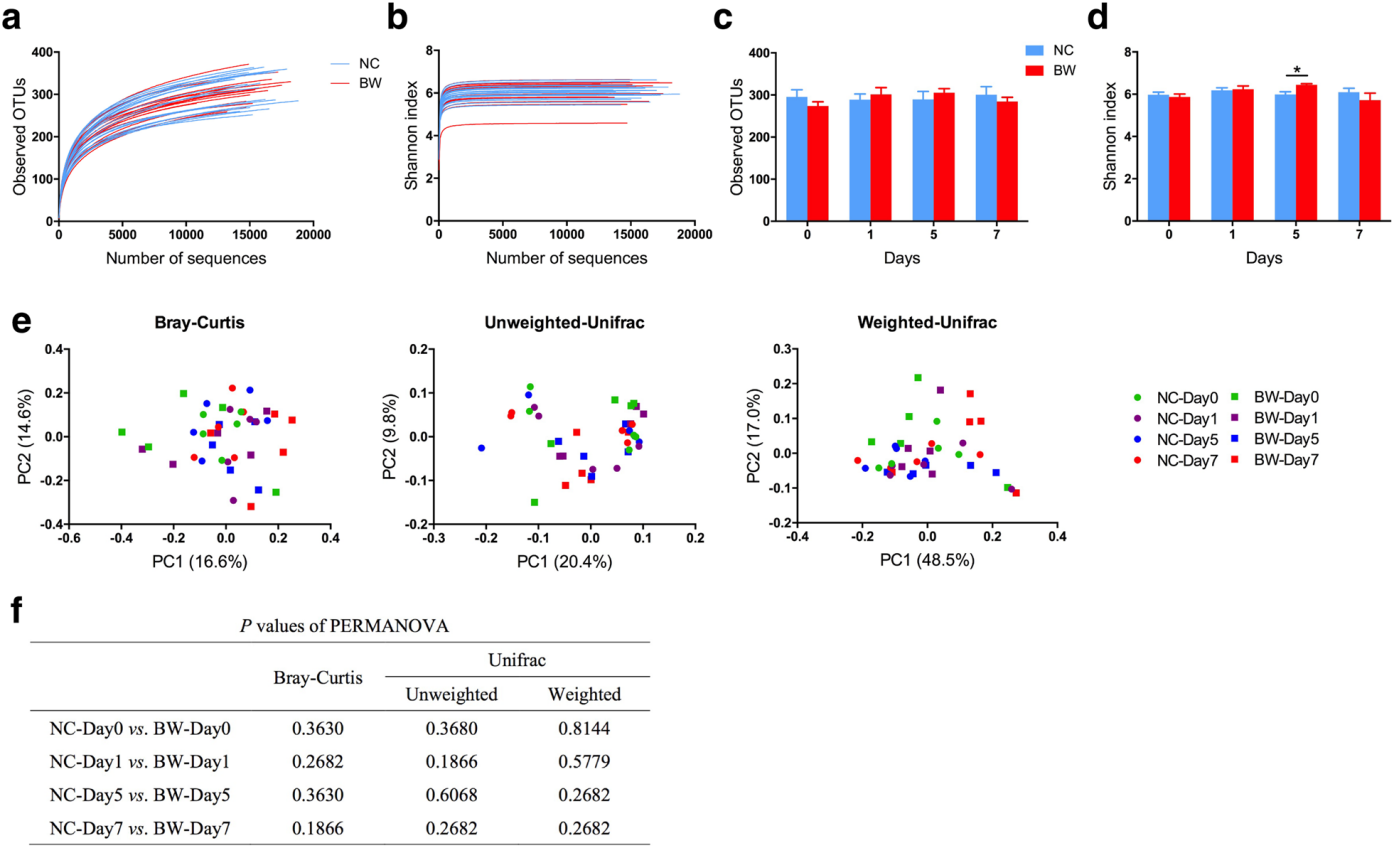
Comparison of gut microbiota with or without *B. wadsworthia* inoculation. **a**, **b** Rarefaction curves and Shannon diversity curves for each sample, respectively. **c**, **d** Rarefaction index and Shannon index respectively of NC and BW group on different days, at sampling depth of 10,810 reads. Data are shown as mean ± S.E.M. and tested by independent samples *t* test. *P < 0.05. **e** Structural comparison of gut microbiota of mice in two groups on Day 0, 1, 5 and 7 based on Bray–Curtis distance, Unweighted Unifrac distance and Weighted Unifrac distance. **f** *P* values of PERMANOVA, *P* values were adjusted with FDR. For both groups, n = 5

The richness of gut microbiota of the experimental mice was estimated by rarefaction, and the diversity was estimated by Shannon diversity index. The shape of the rarefaction curves indicated that new phylotypes would be expected with additional sequencing, while, the Shannon diversity index curves of all samples reached plateaus with the current sequencing depth, suggesting that most diversity had already been captured (Fig. 5a, b).

Comparison of α-diversity of the bacterial communities on Day 0, 1, 5 and 7 exhibited no great difference of the community diversity between BW and NC group (Fig. 5c, d). During the whole treatment, the gut microbiota of the two groups contained the similar rarefaction and Shannon diversity index, only with the exception that, on Day 5, the Shannon index of BW mice was higher than NC mice (P < 0.05), while this tendency disappeared on Day 7.

To further compare the microbiota composition of *B. wadsworthia* infected mice with that of NC mice, principal coordinate analysis (PCoA) based on Bray–Curtis distance, Unweighted and Weighted UniFrac matrices were all performed. Unfortunately, neither of the three plot patterns exhibited divergence of the two groups on different time points (Fig. 5e). PERMANOVA test based on the three distances revealed that on either four time points, the *P* values were all higher than 0.05 (Fig. 5f), which confirmed that the microbiota composition of mice inoculated with *B. wadsworthia* didn’t significantly shift compared with NC group. We also conducted taxon-based analysis at different taxonomic levels (from phylum to genus) by Mann–Whitney U test, while all the *P* values were greater than 0.05 after adjusted with FDR, which implied no significant difference was observed between the two groups (data not shown). Among the 539 OTUs identified in this study, one OTU was assigned as *B. wadsworthia*, which only presented in three samples in BW group. In those three samples, only 2, 12 and 3 sequences were found, respectively. Given the 613,626 quality-filtered sequences totally, the relative abundance of *B. wadsworthia* was too low, even undetectable in most samples. Thus, though ABB agar plating showed *B. wadsworthia* was enriched in BW group of mice due to inoculation, their abundance in the whole microbiota was still lower than the detection limit of the sequencing system.

## Discussion

*Bilophila wadsworthia* belongs to the *Desulfovibrionaceae* family and is the second most recovered isolate of sulfidogenic bacteria in human gut. Sulfidogenic bacteria metabolize sulfated compounds and produce hydrogen sulfide that triggers direct inflammation, exerts genotoxic and cytotoxic effects on epithelial cells, and impairs gut barrier [22]. Correlation of sulfidogenic bacteria to the etiology of chronic metabolic diseases such as obesity [23], type 2 diabetes [24] and colorectal cancer [6] has been explored only recently.

Though *B. wadsworthia* have been identified from a variety of intra-abdominal infections, including sepsis, liver abscesses, cholecystitis et al. [25, 26], the direct evidence linking *B. wadsworthia* with chronic diseases is still insufficient. In the present study described here, we investigated the outcome of *B. wadsworthia* infection on SPF C57BL/6 mice. We hypothesize that increased numbers of *B. wadsworthia* in gut microbiota induces systemic inflammation and thus contributes to the onset of the chronic diseases.

After inoculation, we found that the *B. wadsworthia* strain couldn’t be detected from fresh fecal samples of mice with only one time oral gavage. This refers to a typical microbial ecology phenomenon termed colonization resistance (CR), which is a mechanism that the robust commensal microbiota of the host protects itself against incursion by exogenous and often pathogenic microorganisms [27]. Only in animals whose CR was impaired by antibiotic treatments or in germ-free animals without any microbiota, the hosts showed susceptible to invading pathogens even at a very low dosage [28]. To solve this problem, we repeated exposure of *B. wadsworthia* to mice for 1 week to ensure enough number of live *B. wadsworthia* cells in the gut. We observed that this strain could survive through the digestive tract as live *B. wadsworthia* could be recovered from fresh feces of mice. Such a strategy has been used in many studies to increase the adaptability of the inoculated bacterium to experimental SPF mice [2, 19].

It has been proved that milk-derived or lard-based high saturated fat diet [5, 29] and even an animal-based diet [30] can markedly promote the flourish of *B. wadsworthia* in gut. Saturated fat diet leads to increased hepatic taurine conjugation of bile acids, thus provides more sulfur-rich taurocholic acid in gut, which accelerates the growth of the sulfite-reducing bacterium *B. wadsworthia*. In this study, all the mice were fed with normal chow diet, that didn’t favor the growth of *B. wadsworthia* in gut. This may also contribute to the difficulty to adapt this strain to gut of mice.

In the present study, we observed that *B. wadsworthia* could induce pathological responses to the SPF mice. Seven days of inoculation resulted in a significant decrease of body weight and three main fat mass of BW mice, compared with NC mice. While, the weight of spleen and liver of BW mice showed a reverse trend that animals in this group developed demonstrable hepatosplenomegaly, which is a common clinico-pathological sign related with various infections [31–33].

We also examined the response of colon after *B. wadsworthia* inoculation. All animals had normal colonic morphology and no obvious histologic changes were observed. McOrist et al. also reported that *B. wadsworthia* infected pigs displayed no or minor specific lesions in the small and large intestine [34]. The relative gene expression of pro-inflammatory cytokines IL-6, TNF-α and TLR4 in colon of BW mice displayed similar levels with those of NC mice, suggesting no local immunopathological response induced in colon by *B. wadsworthia*. A previous work has presented that consumption of milk-derived fat not only promoted the expansion of *B. wadsworthia* in SPF mice, but also increased incidence of colitis in colon of genetically susceptible *Il10* ^−/−^ mice, while not in wild-type mice [5]. Our result also demonstrated that the *B. wadsworthia* infection in normal SPF mice cannot directly trigger immune response in colon in the present short-term experiment.

Notably, we observed that *B. wadsworthia* infection provoked a systemic inflammatory response in SPF mice. The key circulating inflammatory cytokines like SAA and IL-6 significantly increased after *B. wadsworthia* inoculation. SAA proteins are acute phase proteins mainly secreted by the liver in response to pro-inflammatory stimuli [35], and their increased levels in serum have been associated with several chronic inflammation-based diseases, such as obesity [36, 37], hyperglycemia [38], insulin resistance [39] and cardiovascular disease [40, 41]. SAA has also been reported to be involved in LPS signaling pathway that links inflammation to metabolic disorders in mice, for instance, de Oliveira et al. found that high fat diet induced metabolic endotoxaemia, and, body weight gain and insulin resistance could be prevented by an SAA-targeted antisense oligonucleotide treatment [42]. IL-6 also represents a keystone cytokine in infection and inflammation [43], which elicits cellular immune responses to affected cells and mucosal humoral responses directed against reinfection. Increasing serum levels of IL-6 provides the basis for the amplification step of chronic inflammatory proliferation [44]. We also detected the levels of serum LBP and TNF-α, their levels in BW group tended to be higher than NC group, while the differences were not statistically significant. Similar phenomenon has also been reported in both humans and rats, that the levels of IL-6 and TNF-α were in parallel with the abundance change of *Bilophila wadsworthia* [8, 45].

As a Gram-negative bacterium, *B. wadsworthia* can release lipopolysaccharide as endotoxin, though its endotoxic activity is relatively low compared with *E. coli* [46]. This strain can also induce procoagulant, and an in vitro study implied its ability to attach to human epithelial cells of the colon, that is usually considered as the first step in establishing infection in the host [46, 47]. Besides, *B. wadsworthia* participates in taurine respiration in human gut which leads to sulfite formation [48]. The metabolic end-product of dissimilatory sulphate and sulfite reduction, hydrogen sulfide, is well documented to be pro-inflammatory and toxic to mucosal tissue at higher physiological doses [49]. These features of *B. wadsworthia* may all contribute to its inflammatory responses and pathogenicity.

Recent years, accumulating evidence points toward the interplay between the microbiota and the immune system as central to the development of chronic metabolic diseases [50–52]. The feature that the outgrowth of *B. wadsworthia* in gut microbiota can promote low-grade systemic inflammation may account a part for the high prevalence of this bacterium in stool of mice with chronic disease such as colon cancer, colitis and low-fat/high-sugar diet induced obese reported previously [5, 6, 8]. Though the *B. wadsworthia* strain used in this study was isolated only from one LADA patient, and its prevalence in other patients is unclear, the data showed here provided important clue for the physiology of this species of commensal bacteria. More patients will be recruited in future study to further confirm the results.

To investigate whether the etiology of *B. wadsworthia* is related to its perturbation of the host gut microbiota, we compared the composition of gut microbiota in mice with and without *B. wadsworthia* infection. It was demonstrated that *B. wadsworthia* made no significant change to the whole existing gut microbiota, in terms of not only the α-diversity but also the OTUs composition. This result indicates that, after 7 days of *B. wadsworthia* infection, the inflammatory phenotypes developed in mice might be caused by *B. wadsworthia* itself and/or its metabolic products, without marked modification of the host’s overall microbiota. Similar results have been reported in several other studies. For example, *A. muciniphila* is a mucin-degrading bacterium that resides in the human gut [53], its abundance decreases in obese and type 2 diabetic mice. Four weeks of *A. muciniphila* administration could reverse high-fat diet-induced metabolic disorders in C57BL/6 mice, without modifying gut microbiota composition [2]. A butyrate-producing bacterium, *Anaerostipes hadrus* BPB5, could increase the butyrate content in healthy SPF C57BL/6 mice after 7 days treatment, while the whole gut microbiota didn’t change significantly [19].

Our results add new evidence to the virulence of *B. wadsworthia*. The decreased body weight and fat mass, apparent hepatosplenomegaly and elevated serum levels of SAA and IL-6 demonstrated that this bacterium triggers the systemic low-grade inflammation of the host. Unfortunately, there is no diabetes mellitus specific parameters addressed in this report. Our next plan is to examine the clinical outcomes of *B. wadsworthia* including glucose and insulin metabolism, to comprehensively understand the metabolic activities and functions of it.

## Conclusions

This report provides a snapshot for responses of *B. wadsworthia* infection in normal mice, which shows the link of this bacterium to the systemic inflammation of the host. Considering that the low-grade inflammation is a common pathogenetic denominator in different metabolic diseases such as obesity, diabetes and aging, to name just a few, our study also implies the potential roles of *B. wadsworthia* to the chronic inflammation related metabolic disorders.

## Abbreviations

ABB: anaerobe basal broth
*B. wadsworthia*: *Bilophila wadsworthia*
Gapdh: glyceraldehyde-3-phosphate dehydrogenase
IL-6: interleukin-6
LADA: latant autoimmune diabetes in adults
LBP: Lipopolysaccharide Binding Protein
OTU: Operational Taxonomic Unit
SAA: Serum Amyloid A
SPF: specific-pathogen-free
TLR-4: Toll-like Receptor-4
TNF-α: tumor necrosis factor-alpha
Tpa: taurine:pyruvate aminotransferase
